# Perioperative Outcomes of Non-Intubated Versus Intubated Anesthesia in Video-Assisted Thoracoscopic Surgery for Early-Stage Non-Small Cell Lung Cancer: A Propensity Score-Matched Analysis

**DOI:** 10.3390/jcm14103466

**Published:** 2025-05-15

**Authors:** Hsiang-Han Huang, Li-Hua Chen, Hou-Chuan Lai, Zhi-Fu Wu, Ching-Lung Ko, Kai-Li Lo, Go-Shine Huang, Wei-Cheng Tseng

**Affiliations:** 1 Department of Anesthesiology, Tri-Service General Hospital, National Defense Medical Center, Taipei 114, Taiwan; alan3413123@gmail.com (H.-H.H.); cool369190@msn.com (L.-H.C.); m99ane@gmail.com (H.-C.L.); aneswu@gmail.com (Z.-F.W.); clko1168@gmail.com (C.-L.K.); kelly8014@gmail.com (K.-L.L.); kshgodoc@gmail.com (G.-S.H.); 2 Graduate Institute of Public Health, National Defense Medical Center, Taipei 114, Taiwan; 3 Department of Anesthesiology, Kaohsiung Medical University Hospital, Kaohsiung Medical University, Kaohsiung 807, Taiwan; 4 Department of Anesthesiology, Faculty of Medicine, College of Medicine, Kaohsiung Medical University, Kaohsiung 807, Taiwan; 5 Center for Regional Anesthesia and Pain Medicine, Wan Fang Hospital, Taipei Medical University, Taipei 116, Taiwan; 6 Graduate Institute of Life Sciences, National Defense Medical Center, Taipei 114, Taiwan

**Keywords:** double-lumen tube, intubated anesthesia, laryngeal mask airway, non-intubated anesthesia, non-small cell lung cancer, video-assisted thoracoscopic surgery

## Abstract

**Background:** Previous studies have shown that ventilation strategies used in general anesthesia influence perioperative outcomes of video-assisted thoracoscopic surgery (VATS). This study investigated the perioperative effects of non-intubated anesthesia (NIA) versus intubated anesthesia (IA) in patients with early-stage non-small cell lung cancer (NSCLC) undergoing VATS. **Methods:** This retrospective cohort study analyzed patients who underwent elective VATS for early-stage NSCLC between January 2015 and December 2022. Patients were categorized into the NIA and IA groups based on the ventilation strategies during general anesthesia. Comprehensive outcome data, including intraoperative and postoperative variables, were compared between the two groups. Univariate and multivariate logistic regression models were used to assess the odds ratios for conversion from NIA to IA. **Results:** A total of 372 patients who received NIA and 1560 who received IA for VATS were eligible for analysis. After propensity score matching, 336 patients were included in each group. In the matched analysis, patients who received NIA demonstrated favorable perioperative outcomes, including reduced opioid consumption, lower postoperative complication rates, and shorter hospital stays, compared to those who received IA. Additionally, patients with a lower baseline oxygen saturation and those who experienced intraoperative pulmonary and cardiovascular adverse events had a higher risk of conversion from NIA to IA. **Conclusions:** NIA during VATS in patients with early-stage NSCLC was associated with superior perioperative outcomes. Prospective studies are warranted to further evaluate the impact of NIA on perioperative outcomes in this patient population.

## 1. Introduction

Lung cancer is the leading cause of cancer-related mortality worldwide [1,2], and non-small cell lung cancer (NSCLC) accounts for approximately 85% of primary pulmonary malignancies [3]. For early-stage NSCLC, surgical resection remains the preferred treatment [1,3]. Compared to open thoracotomy, video-assisted thoracoscopic surgery (VATS) has been a widely adopted minimally invasive technique for early-stage NSCLC, offering advantages such as reduced postoperative pain, shorter chest tube duration and hospital stays, faster recovery, and improved survival rates [4,5].

Traditionally, VATS is performed under general anesthesia (GA) with endobronchial intubation to facilitate one-lung ventilation (OLV) and optimize surgical exposure [6]. However, intubated anesthesia (IA) during VATS carries risks, including airway trauma, ventilator-associated lung injury, residual neuromuscular blockade, and postoperative nausea and vomiting (PONV) [7,8]. With the growing emphasis on Enhanced Recovery after Surgery (ERAS) protocols, non-intubated anesthesia (NIA) for VATS has gained popularity in recent decades. It is characterized by maintaining patients’ spontaneous breathing during surgery while keeping them fully alert, sedated, or under GA [9]. A previous meta-analysis also demonstrated that NIA in VATS was associated with a shorter hospital stay compared to IA [10]. Despite these potential benefits, concerns about patient safety remain, particularly in patients with lung cancer.

To the best of our knowledge, only a few observational studies with limited sample sizes have compared NIA and IA during VATS in terms of perioperative outcomes in patients with early-stage NSCLC. To examine the hypothesis that NIA during VATS may improve perioperative outcomes, we conducted a retrospective cohort analysis comparing intraoperative and postoperative profiles between NIA and IA in early-stage NSCLC patients undergoing VATS. This study aimed to evaluate the impact of NIA on perioperative outcomes and to identify risk factors associated with unintended conversion to IA during VATS in patients with early-stage NSCLC.

## 2. Materials and Methods

### 2.1. Study Design and Setting

This retrospective cohort study was conducted at Tri-Service General Hospital (TSGH), Taipei, Taiwan (Republic of China).

### 2.2. Participants and Data Sources

This retrospective study was approved by the ethics committee of TSGH, which waived the need for informed consent (TSGHIRB No: 1-107-05-008). Relevant data were retrieved from medical records and the electronic database at TSGH for patients classified as American Society of Anesthesiologists (ASA) I to III who underwent elective VATS for tumor–node–metastasis (TNM) stage 0 (carcinoma in situ) to II NSCLC between January 2015 and December 2022. Patients included in the study received either NIA or IA, based on a collaborative decision between the anesthesiologist and surgeon, taking into account the patient’s medical history, preoperative findings, and surgical preferences. Exclusion criteria were non-NSCLC histology, advanced-stage disease (III to IV), ASA class IV or higher, age below 20 years, emergency surgery, and incomplete data. In addition, patients who underwent conversion to open thoracotomy were excluded during cohort selection to ensure comparability between minimally invasive approaches and to clarify the impact of the anesthesia modality.

### 2.3. Anesthetic Technique

Upon arrival in the operating room, standard monitoring, including electrocardiography (lead II), noninvasive blood pressure, and pulse oximetry, was established for all patients. Additionally, bispectral index (BIS) monitoring (BIS^TM^ Complete 2-Channel Monitor; Medtronic, Minneapolis, MN, USA) was applied to assess anesthetic depth. Hemodynamic values and BIS data were recorded every 5 min. Radial arterial blood pressure monitoring was implemented when clinically indicated. No premedication was administered before anesthesia induction. After preoxygenation, GA was induced with fentanyl, 2% lidocaine, and propofol in all patients.

When patients lost consciousness, either cisatracurium or rocuronium was administered to facilitate endobronchial intubation with a left-sided double-lumen tube (DLT; Shiley^TM^ Endobronchial Tube, Mallinckrodt Medical, Athlone, Ireland) in the IA group. After confirming correct tracheal intubation by capnography, the optimal insertion depth was initially determined using a formula from our institute [11], and the position of the DLT was then adjusted using fiberoptic bronchoscopy. Conversely, in the NIA group, a laryngeal mask airway (LMA; LMA Classic^TM^, Teleflex Medical, Westmeath, Ireland) was used to prevent airway collapse after loss of consciousness, and no neuromuscular blocking agents were administered to maintain spontaneous breathing [12]. Capnography was also applied to confirm and optimize LMA placement. Before the placement of DLT or LMA, intravenous dexamethasone (5 mg) was routinely administered in the absence of contraindications. Patients were subsequently positioned in the lateral decubitus position according to the surgical site, and the correct placement of the DLT or LMA was reconfirmed.

GA was maintained using a target-controlled infusion (TCI) pump (Orchestra^®^ Base Primea, Fresenius Kabi AG, Bad Homburg, Germany) with propofol at an effect-site concentration (Ce) of 3–4 mcg/mL in a fraction of inspired oxygen (FiO_2_) of 50–100% at a flow rate of 1000 mL/min, a sevoflurane vaporizer set between 1% and 5% in 50–100% oxygen at the same flow rate within a closed breathing system, or a mixed anesthetic technique combining both approaches. OLV was initiated 10 min before surgery in the IA group. During OLV, patients’ lungs were ventilated using the volume control mode with a tidal volume (V_T_) of 4–6 mL/kg of predicted body weight and a positive end-expiratory pressure (PEEP) of 5–10 cmH_2_O, while maintaining a peak airway pressure below 35 cmH_2_O [13]. The level of end-tidal carbon dioxide (EtCO_2_) was maintained at 35–45 mmHg by adjusting the ventilation rate. According to hemodynamic responses, the Ce of propofol in the TCI pump and sevoflurane concentration were titrated in increments of 0.2–0.5 mcg/mL and 0.5–1%, respectively. Additional bolus doses of fentanyl and either cisatracurium or rocuronium were administered as needed during surgery.

For patients in the NIA group, conversion to IA with OLV was performed in cases of hemodynamic instability, intractable hypoxemia or hypercapnia, inadequate ventilation, significant patient movement interfering with surgery, or difficulty in controlling bleeding. After the decision for conversion, a single-lumen endotracheal tube was inserted following bolus administration of rocuronium (0.6 mg/kg). A bronchial blocker was then placed under fiberoptic bronchoscopic guidance while the patient remained in the lateral decubitus position.

Postoperatively, all patients were transferred to either the post-anesthesia care unit (PACU) or the intensive care unit (ICU) for postoperative observation and care. Patients monitored in the PACU were discharged once they demonstrated stable vital signs and acceptable pain scores (numeric rating scale [NRS] ≤ 4). Intravenous tramadol (50–100 mg) and droperidol (1.25 mg) were administered as required for analgesic rescue and PONV, respectively.

### 2.4. Surgical Procedure

After the patient was positioned in the lateral decubitus position with flexion of the operating table to widen the intercostal space (ICS), VATS was performed using two to three ports, based on the surgeon’s preference, experience, and procedural requirements. The thoracoscopic port for a 30°-angled camera was placed at the 7th or 8th ICS along the mid-axillary line. A 4–5 cm working window was created at the 5th or 6th ICS in the mid-clavicular line. If necessary, an additional port was made at the 7th ICS in the post-scapular line.

The incision made on the chest wall induced iatrogenic pneumothorax, leading to subsequent lung collapse on the surgical side. After accessing the thoracic cavity, the surgeon performed an intercostal block from the 3rd to 8th intercostal nerves using 1 mL of 0.5% bupivacaine per nerve. At the end of surgery, a chest tube with a drainage device (Pacific Hospital Supply Co., Ltd., Miaoli County, Taiwan) was inserted. Standard postoperative care was provided, and the chest tube was removed once air leakage ceased and drainage amount was less than 100 mL/day.

### 2.5. Variables

Patient data were retrospectively collected, including ventilation strategy, time since the earliest included patient as a surrogate for the calendar year, calendar period, sex, age at the time of surgery, body habitus, smoking and alcohol consumption, preoperative pulmonary function (forced expiratory volume in 1 s [FEV_1_] and diffusing capacity of the lung for carbon monoxide [DLCO]), ASA physical status, Mallampati score, histological subtype, lesion site, TNM stage, differentiation grade, procedural type, anesthetic technique, and clinical experience of the surgeon and anesthesiologist.

The Charlson comorbidity index (CCI), ranging from 0 to 37 (indicating least to highest comorbidity burden), was considered in evaluating perioperative risks, particularly in NSCLC patients with multiple comorbidities [14]. Additionally, preoperative functional status was assessed in metabolic equivalents (METs), with patients stratified into two groups: ≥ 4METs and <4 METs. This classification was based on evidence that patients with a functional capacity of <4 METs during daily activities exhibited an increased risk of perioperative cardiac events and long-term complications [15].

Intraoperative hemodynamic parameters (mean blood pressure [MBP], heart rate [HR], and oxygen saturation [SpO_2_]) were recorded at selected time points for analysis. Moreover, surgery- and anesthesia-related variables were documented, including anesthetic induction and emergence time, total duration of surgery and anesthesia, intraoperative fentanyl consumption, use of epidural analgesia and NSAIDs, volume of fluid intake and blood loss, need for intraoperative blood transfusion, and incidence of adverse events during surgery. The induction time was defined as the period from the administration of anesthetic agents for induction to the completion of LMA or DLT placement; the emergence time was defined as the period from the end of surgery to the recovery of consciousness. Pulmonary adverse events during surgery included hypoxemia (SpO_2_ < 90%), hypercapnia (EtCO_2_ > 60 mmHg), and bronchospasm, whereas cardiovascular adverse events consisted of hypotension (MBP < 60 mmHg), arrhythmias, and myocardial ischemia/infarction. The requirement for unintended conversion from NIA to IA and its underlying causes were also recorded.

Postoperative data collection included the grade of surgical complications according to the Clavien–Dindo classification, ranging from grade 0 (no complication) to grade V (death). Other postoperative variables, such as fentanyl consumption, adverse events, need for postoperative mechanical ventilation and ICU admission, length of hospital stay, patient and surgeon satisfaction with anesthesia (1 = very unsatisfactory, 2 = unsatisfactory, 3 = neutral, 4 = satisfactory, 5 = very satisfactory), and healthcare costs, were also documented. Postoperative pulmonary complications included atelectasis, pneumonia, and prolonged air leak, whereas cardiovascular complications comprised hypotension (MBP < 60 mmHg), arrhythmias, and myocardial ischemia/infarction.

### 2.6. Statistical Methods

The primary outcomes involved perioperative parameters, including hemodynamic stability, duration of surgery and anesthesia, opioid consumption, analgesic use, fluid intake and blood loss, transfusion requirement, incidence of adverse events, and surgical complications, compared between the NIA and IA groups. The secondary outcomes were assessed through variables such as the length of hospital stay, anesthesia satisfaction, and healthcare costs. All data were presented as mean ± standard deviation (SD) or as numbers with percentages. Patient characteristics and perioperative parameters were compared between the two groups, which received different ventilation strategies, using Student’s *t*-test or the Chi-squared test. Subgroup analyses of perioperative complications (pulmonary and cardiovascular complications) by calendar period, surgeon experience, procedural type and anesthesia technique were conducted using logistic regression models between the two ventilation strategies. Interaction effects between ventilation strategies and these stratification variables were also assessed. The association between the aforementioned variables and unintended conversion from NIA to IA was analyzed using logistic regression, both with and without adjustment. Conversion to IA was compared across patient characteristics and perioperative parameters using a univariate logistic regression model, followed by a multivariate logistic regression model. Variables that demonstrated statistical significance in the univariate analysis were included in the multivariate analysis for further adjustment. Statistical significance was set at *p* value < 0.05.

Propensity score (PS) matching was performed using IBM SPSS Statistics (version 23.0; IBM SPSS Inc., Chicago, IL, USA) to achieve comparability between the two ventilation strategies before surgery. The most similar PS values for preoperative variables were matched in a 1:1 ratio without replacement between the NIA and IA groups, with a caliper width of 0.2 SD of the logit of the PS. Preoperative variables used for PS matching included time since the earliest included patient, sex, age, body mass index (BMI), smoking and alcohol consumption, CCI, preoperative pulmonary function, ASA class, Mallampati score, histological subtype, lesion site, TNM stage, differentiation grade, surgeon and anesthesiologist experience, procedural type, and anesthetic technique. Calendar period and functional status were excluded from the matching process to enhance the rigor of PS matching, as they were highly correlated with time since the earliest included patient and ASA class, respectively. Covariate balance was assessed using standardized mean difference (SMD; <0.1 is considered acceptable).

## 3. Results

**Figure 1:**
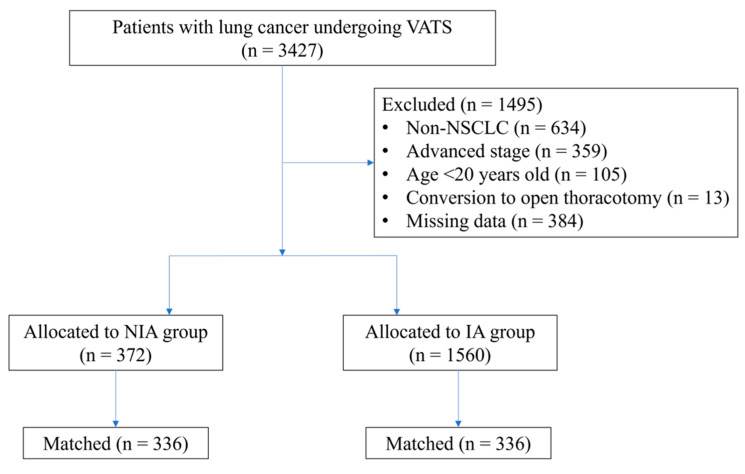
Flow diagram detailing the selection of patients included in the retrospective analysis. IA, intubated anesthesia; NIA, non-intubated anesthesia; NSCLC, non-small cell lung cancer; VATS, video-assisted thoracoscopic surgery.

A total of 3427 patients undergoing VATS for lung cancer were initially screened for the study. After applying the exclusion criteria, 1932 eligible patients remained in the final cohort for analysis. Among them, 372 patients received NIA, while 1560 received IA. The patient selection process and group distribution are detailed in Figure 1.

### 3.1. Patient and Treatment Characteristics

Patient and treatment characteristics are presented in Table 1. Significant differences between the NIA and IA groups were observed in multiple variables, including the time since the earliest included patient, calendar period, sex, age, BMI, smoking status, CCI, ASA class, preoperative functional status, Mallampati score, TNM stage, differentiation grade, surgeon experience, procedural type, and anesthetic technique.

PS matching is a crucial statistical method for minimizing the effect of confounding factors in observational studies [16]. Therefore, logistic regression-derived PS values were applied to adjust for baseline characteristics and treatment selection between the two groups. A total of 336 patient pairs were formed after matching (Figure 1). Significant differences remained between the two matched groups in several patient characteristics and treatment factors.

Both the time since the earliest included patient and the calendar period differed significantly between the two matched groups, indicating a growing trend toward performing NIA in patients with early-stage NSCLC undergoing VATS. Mid-career surgeons were more inclined to adopt NIA (92.5% vs. 75.6%; *p* < 0.001), whereas both younger and highly experienced surgeons were more accustomed to performing VATS under IA. In addition, inhalational anesthetics were more frequently used as the sole anesthetic agent in the matched NIA group compared to the matched IA group (14.6% vs. 5.0%; *p* < 0.001).

### 3.2. Perioperative Outcomes

Perioperative outcomes of VATS are shown in Table 2. Intraoperative variables, including the duration of surgery and anesthesia, anesthetic induction and emergence time, intraoperative opioid consumption, use of epidural analgesia, intraoperative fluid intake and blood loss, need for intraoperative blood transfusion, and incidence of patient movement interfering with the procedure, showed significant differences between the two groups. Postoperative variables also differed significantly between the two groups, including postoperative opioid consumption, incidence of postoperative pulmonary complications, need for postoperative mechanical ventilation and ICU admission, length of postoperative and total hospital stay, patient satisfaction, and total healthcare costs consisting of both surgery- and anesthesia-related expenses.

After PS matching, in terms of surgery- and anesthesia-related time, the matched NIA group demonstrated significantly shorter total anesthesia duration, as well as reduced anesthetic induction and emergence time, compared to the matched IA group. Moreover, patients in the matched NIA group had lower opioid consumption and required a smaller volume of intraoperative fluids than those in the matched IA group. Except for intraoperative patient movement, the incidence of perioperative adverse events, including postoperative pulmonary and cardiovascular complications, was significantly lower in the matched NIA group compared to the matched IA group. Patients in the matched NIA group also had significantly lower requirements for postoperative mechanical ventilation and ICU admission than those in the matched IA group. Ultimately, the matched NIA group exhibited significantly shorter hospital stays, better patient satisfaction, and reduced healthcare costs compared to the matched IA group.

### 3.3. Intraoperative Hemodynamic Stability

**Figure 2:**
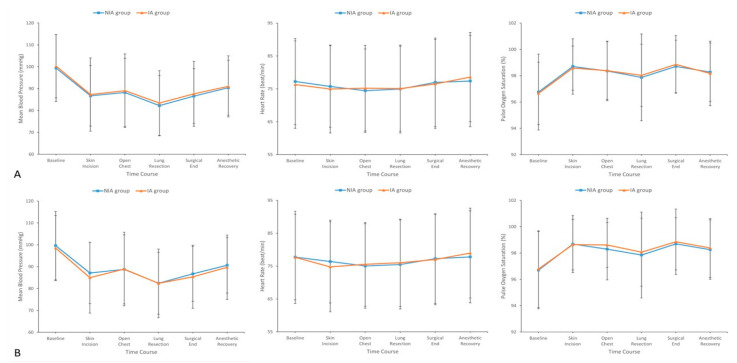
Hemodynamic parameters including mean blood pressure, heart rate, and oxygen saturation at selected time points during surgery between the (**A**) overall and (**B**) matched groups. IA, intubated anesthesia; NIA, non-intubated anesthesia.

The changes in intraoperative hemodynamic parameters, including MBP, HR, and SpO_2_, are illustrated in Figure 2. During surgery, hemodynamic parameters did not significantly differ between the NIA and IA groups at predefined time points (Figure 2A). After PS matching, these parameters remained statistically similar between the matched groups throughout the intraoperative period (Figure 2B).

### 3.4. Subgroup Analyses of Perioperative Complications

Subgroup analyses of perioperative complications are presented in Table 3. These analyses were stratified by calendar period, surgeon experience, procedural type, and anesthesia technique. No interaction effects were observed between ventilation strategy and these factors on perioperative complications. Furthermore, there were no statistically significant differences in perioperative complications between the NIA and IA groups across all stratifications, suggesting comparable patient safety regardless of these variables. However, patient selection and provider expertise may still influence surgical and anesthetic outcomes, warranting further investigation in larger cohorts.

### 3.5. Incidence and Causes of Conversion to IA

**Figure 3:**
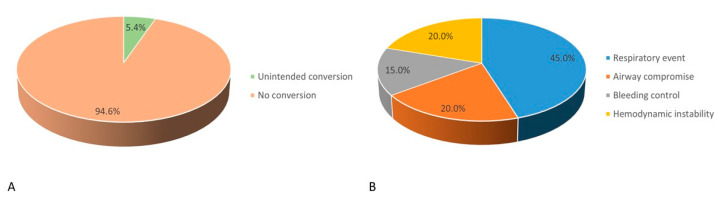
(**A**) Incidence and (**B**) causes of unintended conversion from non-intubated anesthesia to intubation anesthesia.

The incidence of unintended conversion from NIA to IA and its underlying causes are illustrated in Figure 3. In this study, 5.4% (n = 20) of overall patients receiving NIA (n = 372) required conversion to IA during VATS (Figure 3A). The most common cause of conversion was respiratory events (45%, n = 9), including hypoxemia and hypercapnia. Other contributing factors included airway compromise (20%, n = 4), difficulty in controlling bleeding (15%, n = 3), and hemodynamic instability (20%, n = 4) (Figure 3B).

### 3.6. Risk Factors for Conversion to IA

The risk factors for unintended conversion from NIA to IA are shown in Table 4. Patients receiving NIA for VATS with lower baseline SpO_2_ levels had a significantly higher risk of conversion than those with higher SpO_2_ levels (odds ratio [OR], 0.87; 95% confidence interval [CI], 0.78–0.97; *p* = 0.011). After adjusting for differentiation grade and intraoperative pulmonary and cardiovascular adverse events in the multivariate model, lower baseline SpO_2_ remained a significant predictor of conversion (OR, 0.89; 95% CI, 0.80–1.00; *p* = 0.042). Additionally, the presence of intraoperative pulmonary and cardiovascular adverse events was strongly associated with an increased risk of conversion after the multivariate analysis (*p* < 0.001).

## 4. Discussion

This study provided a comprehensive comparison of perioperative outcomes between NIA and IA in patients undergoing VATS for early-stage NSCLC. The findings indicated that adopting NIA in VATS was associated with shorter anesthesia-related durations, reduced opioid consumption, lower fluid intake, and fewer perioperative complications, while maintaining comparable intraoperative hemodynamic stability compared to IA. Additionally, NIA decreased the need for critical care, shortened hospital stays, enhanced patient satisfaction, and lowered healthcare costs.

In our analysis, NIA during VATS exhibited a shorter anesthesia-related duration, including both induction and emergence time, consistent with earlier studies [9,17]. This result was unsurprising due to the nature of NIA, which eliminates the need for endobronchial intubation and reduces the recovery time from neuromuscular blockade. In addition, there was similar surgical duration between the NIA and IA groups as that reported in previous studies [9,18], suggesting that the adoption of NIA had minimal impact on the surgical procedure. A meta-analysis also showed similar findings on anesthesia and surgery time between the NIA and IA groups for VATS in comparison to our study [19]. Notably, a recent retrospective cohort study demonstrated a shorter surgical duration with NIA in VATS [17], which may be attributed to differences in the definition of surgical time between studies.

Regarding intraoperative blood loss, both the recorded volume during surgery and the need for blood transfusion—used as an indirect indicator of surgical bleeding—were documented in our analysis. Although one study reported reduced blood loss and transfusion requirements with NIA compared to IA during VATS [17], our findings demonstrated similar blood loss volumes and transfusion rates between the two groups, as observed in most previous research [18,20,21]. These results suggest that intraoperative blood loss is more likely influenced by factors such as tumor size, extent of pleural adhesions, surgical technique, and surgeon experience, rather than the ventilation strategy applied.

NIA has been associated with less postoperative pain following VATS for various thoracic diseases [9,20]. Furthermore, a previous randomized controlled trial demonstrated that patients receiving NIA required fewer opioid doses than those receiving IA during VATS for spontaneous pneumothorax [21]. As numerical pain scores (e.g., NRS or visual analogue scale) were not consistently available in our cohort, we used intraoperative and postoperative fentanyl consumption as a validated surrogate for perioperative pain intensity. This method has been previously adopted in ERAS protocols as a practical, analgesia-centered indicator of pain burden [22]. Consistent with prior findings, our study revealed that both intraoperative and postoperative opioid consumption were lower in the NIA group compared to the IA group, reflecting a potential decrease in nociceptive input and a smoother recovery profile. The reduction in intraoperative opioid use with NIA may be attributed to the absence of intubation-related stimuli, and gentler surgical manipulation for decreasing the cough reflex. Postoperatively, patients in the IA group may experience higher pain levels due to the effects of intubation and residual neuromuscular blockade, potentially explaining their greater opioid requirements [23]. Therefore, NIA appears to support the objectives of ERAS protocols in thoracic surgery by minimizing opioid use and its relative side effects [22].

Acute lung injury following OLV is a potential concern, with complex and multifactorial pathophysiologic mechanisms. Since OLV is non-physiologic, histologic lung injury and adverse effects may still occur, despite the application of protective ventilation strategies [13]. Compared to IA, NIA during VATS has been shown to reduce the incidence of postoperative pulmonary complications [23], which aligns with our findings and is probably attributed to the attenuation of inflammatory responses induced by OLV. Furthermore, a previous meta-analysis reported that patients receiving NIA for VATS had a lower overall incidence of postoperative complications, including cardiovascular adverse events, than those receiving IA [19]. These findings suggest that avoiding intubation, mechanical ventilation, and neuromuscular blockade may reduce perioperative cardiopulmonary stress by preserving more physiological respiratory mechanics and minimizing hemodynamic fluctuations during thoracic surgery. Given these benefits of NIA, our study demonstrated that NIA during VATS was associated with fewer requirements for postoperative mechanical ventilation and ICU admission, shorter hospital stays, greater patient satisfaction, and lower healthcare costs, which is consistent with results from previous studies [10,17,18,19,20,21,23].

During VATS, intraoperative conversion from NIA to IA to manage complex conditions presents a major challenge for anesthesiologists. In our cohort, we observed a 5.4% conversion rate from NIA to IA, which is consistent with previously reported rates ranging from approximately 1.8% to 11% during VATS [10,24]. The most common reason for conversion is prolonged hypoxemia or hypercapnia, aligning with our findings. Other contributing factors include bleeding, severe adhesions, excessive diaphragmatic movement, and hemodynamic instability [24]. Although the incidence of conversion to IA is relatively low, well-established protocols and essential equipment should be readily available to ensure a timely and effective response. For conversion, either inserting a single-lumen endotracheal tube followed by a bronchial blocker under bronchoscopic guidance or placing a DLT by a sufficiently skilled anesthesiologist in a modified supine rather than a full lateral position is recommended [8]. In our study, a lower baseline SpO_2_ level and the occurrence of intraoperative pulmonary and cardiovascular adverse events were identified as risk factors for conversion from NIA to IA during VATS in patients with early-stage NSCLC. These findings underscore the importance of preoperative screening and intraoperative vigilance in selecting candidates for NIA, thereby enhancing patient safety and supporting individualized ventilation strategies during VATS. However, a retrospective study reported that advanced age and high BMI were significant risk factors for conversion [25], which were not observed in our findings. The discrepancies in identifying high-risk patients may arise from differences in the enrollment period, heterogeneity in patient populations and surgical procedures, and the choice of anesthetic agents. Thus, further studies are warranted to determine risk factors in specific populations.

Appropriate patient selection is essential to prevent significant perioperative crises and potential failure of NIA during VATS. Several patient characteristics have been proposed as recommended contraindications, including higher ASA physical status (ASA class ≥ III), obesity (BMI > 30 kg/m^2^), known or expected difficult airway (Mallampati score III–IV), hemodynamically instability, preexisting severe pulmonary diseases (PaO_2_ ≤ 60 mmHg or PaCO_2_ ≥ 50 mmHg), neurologic disorders, coagulopathy (international normalized ratio > 1.5), and persistent cough or excessive respiratory secretions [8,26]. An increased risk of gastric content regurgitation and the presence of extensive pleural adhesions are also considered contraindications [8]. However, apart from the absence of patient consent, most of these factors remain relative rather than absolute contraindications. Thus, NIA is still feasible in these patients undergoing VATS, although extra caution should be taken during surgery.

NIA for VATS can be performed using various oxygen delivery devices, such as a simple nasal cannula, face mask, or high-flow nasal cannula, in patients who are either awake or sedated. Although these oxygenating methods are less invasive, NIA with formal airway support via a supraglottic airway under GA is believed to reduce patient anxiety and distress, minimize movement and coughing, and provide superior airway control and oxygenation [9]. Therefore, at our institution, GA with an LMA for oxygenation and ventilation is the preferred approach for performing NIA during VATS. Notably, LMA inflation can cause pharyngeal compression, leading to postoperative sore throat in some patients [23]; however, its incidence and severity remain lower compared to a DLT.

The findings of this study offer practical implications for anesthetic decision-making in patients with early-stage NSCLC undergoing VATS, supporting the inclusion of NIA as a viable alternative to IA in appropriately selected cases. Specifically, patients with preserved pulmonary reserve, early-stage disease, and lower surgical complexity (e.g., wedge resection) may benefit most from NIA, which aligns with ERAS principles by minimizing ventilator-induced lung injury, reducing opioid burden, and facilitating faster recovery [27]. In contrast, patients with borderline oxygenation, complex surgical procedures, or anticipated difficult airways may be more safely managed with conventional IA. Guided by preoperative risk stratification and intraoperative contingency planning, our study also provides a framework for individualized anesthetic planning, and reinforces a shift from a “one-size-fits-all” approach toward a personalized anesthetic strategy.

Our study had several limitations. First, it was conducted at a single medical center, and further large-scale, multicenter studies are needed to validate our findings. Second, as a retrospective cohort study, patients were not randomly allocated. Although PS matching was performed to minimize confounding in this observational study, the small group size may affect the reliability of statistical significance. Third, even though multivariate and PS matching analyses were conducted with numerous variables to obtain reliable results, some unmeasured confounding factors could not be excluded and may have influenced the outcomes. Fourth, our study focused on short-term outcomes between NIA and IA for VATS, but long-term outcomes were not assessed. Fifth, our analysis included only patients with an inserted LMA, as it is the most frequently used oxygenating method for NIA during VATS at our institution. However, different oxygen delivery devices may have distinctive effects on perioperative outcomes in patients undergoing VATS. Lastly, our study analyzed only patients diagnosed with NSCLC, which accounts for the majority of primary pulmonary malignancies. Therefore, our findings may not be directly applicable to patients with non-NSCLC or other thoracic diseases. Despite these limitations, our results may have important clinical implications for managing NIA during VATS in patients with early-stage NSCLC.

## 5. Conclusions

Compared to IA, the application of NIA during VATS in patients with early-stage NSCLC was associated with favorable perioperative outcomes, including reduced opioid use, lower postoperative complication rates, and shorter hospital stays. Additionally, patients with preexisting respiratory compromise or intraoperative complications may be at high risk for conversion from NIA to IA. Consequently, NIA is a safe, feasible and effective anesthetic technique for VATS and should be integrated into ERAS protocols in thoracic surgery.

## Figures and Tables

**Table 1:** Patient and treatment characteristics for the overall group and matched group after propensity scoring.

Variables		Overall Patients			Matched Patients			
		NIA Group (n = 372)	IA Group (n = 1560)	*p* Value	NIA Group (n = 336)	IA Group (n = 336)	*p* Value	SMD
Time since the earliest included patient (yrs), mean (SD)		5.03 (2.25)	4.40 (2.37)	<0.001	4.93 (2.30)	3.32 (2.55)	<0.001	0.663
Calendar period, n (%)				<0.001			<0.001	0.331
	2015–2016	49 (13.2)	311 (20.0)		49 (14.6)	130 (38.7)		
	2017–2018	65 (17.5)	400 (25.6)		60 (17.9)	85 (25.3)		
	2019–2020	85 (22.8)	323 (20.7)		78 (23.2)	45 (13.4)		
	2021–2022	173 (46.5)	526 (33.7)		149 (44.3)	76 (22.6)		
Sex (male), n (%)		126 (33.9)	635 (40.7)	0.015	118 (35.1)	106 (31.5)	0.326	0.076
Age (yrs old), mean (SD)		57.92 (11.05)	60.72 (10.81)	<0.001	58.25 (11.14)	58.25 (12.04)	0.997	0.000
BMI (kg/m^2^), mean (SD)		22.62 (2.99)	24.04 (3.96)	<0.001	22.77 (3.02)	22.44 (3.03)	0.156	0.109
Cigarette smoking, n (%)		58 (15.6)	374 (24.0)	<0.001	58 (17.3)	52 (15.5)	0.532	0.049
Alcohol consumption, n (%)		12 (3.2)	75 (4.8)	0.186	11 (3.3)	10 (3.0)	0.825	0.017
Charlson comorbidity index, mean (SD)		3.87 (1.34)	4.28 (1.49)	<0.001	3.92 (1.35)	3.98 (1.39)	0.593	0.044
FEV_1_ (% predicted), mean (SD)		92.05 (16.92)	90.39 (17.96)	0.105	91.66 (17.16)	90.67 (18.04)	0.463	0.056
DLCO (% predicted), mean (SD)		95.67 (17.71)	96.02 (18.20)	0.739	94.92 (17.26)	95.95 (19.18)	0.463	0.056
ASA class, n (%)				<0.001			0.472	0.047
	I	15 (4.0)	37 (2.4)		12 (3.6)	16 (4.8)		
	II	289 (77.7)	1089 (69.8)		261 (77.6)	248 (73.8)		
	III	68 (18.3)	434 (27.8)		63 (18.8)	72 (21.4)		
Functional status (≥4 METs), n (%)		301 (80.9)	1106 (70.9)	<0.001	270 (80.4)	262 (78.0)	0.447	0.059
Mallampati score, n (%)				0.001			0.228	0.066
	I	4 (1.1)	5 (0.3)		3 (0.9)	3 (0.9)		
	II	356 (95.7)	1427 (91.5)		321 (95.5)	328 (97.6)		
	III	12 (3.2)	128 (8.2)		12 (3.6)	5 (1.5)		
Histological subtype, n (%)				0.326			0.590	0.040
	Adenocarcinoma	270 (72.6)	1182 (75.8)		242 (72.0)	238 (70.8)		
	SCC	101 (27.1)	370 (23.7)		93 (27.7)	95 (28.3)		
	Large cell carcinoma	1 (0.3)	8 (0.5)		1 (0.3)	3 (0.9)		
Lesion site, n (%)				0.950			0.810	0.049
	RUL	112 (30.1)	495 (31.7)		100 (29.8)	107 (31.9)		
	RML	32 (8.6)	132 (8.5)		29 (8.6)	32 (9.5)		
	RLL	80 (21.5)	321 (20.6)		73 (21.7)	74 (22.0)		
	LUL	86 (23.1)	353 (22.6)		79 (23.5)	66 (19.6)		
	LLL	62 (16.7)	256 (16.4)		55 (16.4)	57 (17.0)		
	Lingula	0 (0.0)	3 (0.2)		0 (0.0)	0 (0.0)		
TNM stage, n (%)				<0.001			0.177	0.072
	0 (CIS)	45 (12.1)	64 (4.1)		39 (11.6)	25 (7.4)		
	I	239 (64.2)	1075 (68.9)		210 (62.5)	223 (66.4)		
	II	88 (23.7)	421 (27.0)		87 (25.9)	88 (26.2)		
Differentiation grade, n (%)				0.005			0.851	0.022
	I	94 (25.3)	282 (18.1)		84 (25.0)	83 (24.7)		
	II	201 (54.0)	891 (57.1)		182 (54.2)	177 (52.7)		
	III	77 (20.7)	387 (24.8)		70 (20.8)	76 (22.6)		
Surgeon experience (yrs), n (%)				<0.001			<0.001	0.234
	<10	13 (3.5)	241 (15.4)		13 (3.9)	34 (10.1)		
	10–20	345 (92.7)	1185 (76.0)		311 (92.5)	254 (75.6)		
	>20	14 (3.8)	134 (8.6)		12 (3.6)	48 (14.3)		
Procedural type, n (%)				<0.001			0.507	0.045
	Wedge resection	339 (91.1)	768 (49.2)		303 (90.2)	308 (91.7)		
	Segmentectomy	21 (5.7)	334 (21.4)		21 (6.2)	21 (6.2)		
	Lobectomy	12 (3.2)	458 (29.4)		12 (3.6)	7 (2.1)		
Anesthesiologist experience (yrs), n (%)				0.568			0.647	0.036
	<10	46 (12.4)	226 (14.5)		42 (12.5)	43 (12.8)		
	10–20	276 (74.2)	1133 (72.6)		252 (75.0)	243 (72.3)		
	>20	50 (13.4)	201 (12.9)		42 (12.5)	50 (14.9)		
Anesthesia technique, n (%)				<0.001			<0.001	0.160
	TIVA	55 (14.8)	626 (40.1)		54 (16.1)	61 (18.2)		
	INHA	50 (13.4)	39 (2.5)		49 (14.6)	17 (5.0)		
	Mixed anesthesia	267 (71.8)	895 (57.4)		233 (69.3)	258 (76.8)		

Notes: Data shown as mean ± SD or n (%). ASA, American Society of Anesthesiologists; BMI, body mass index; CIS, carcinoma in situ; DLCO, diffusing capacity of the lung for carbon monoxide; FEV_1_, forced expiratory volume in 1 s; IA, intubated anesthesia; INHA, inhalational anesthesia; LLL, left lower lobe; LUL, left upper lobe; MET, metabolic equivalent; NIA, non-intubated anesthesia; RLL, right lower lobe; RML, right middle lobe; RUL, right upper lobe; SCC, squamous cell carcinoma; SD, standard deviation; SMD, standardized mean difference; TIVA, total intravenous anesthesia; TNM, tumor–node–metastasis.

**Table 2:** Perioperative outcomes for the overall group and matched group after propensity scoring.

Variables		Overall Patients			Matched Patients			
		NIA Group (n = 372)	IA Group (n = 1560)	*p* Value	NIA Group (n = 336)	IA Group (n = 336)	*p* Value	SMD
**Intraoperative variables**								
Operation time (mins), mean (SD)		74.46 (24.63)	103.60 (85.03)	<0.001	74.88 (25.04)	79.58 (62.28)	0.200	NA
Anesthesia time (mins), mean (SD)		102.03 (28.33)	143.91 (91.74)	<0.001	102.38 (28.96)	116.90 (67.96)	<0.001	NA
Induction time (mins), mean (SD)		20.50 (8.66)	30.05 (13.43)	<0.001	20.35 (8.66)	27.55 (12.50)	<0.001	NA
Emergence time (mins), mean (SD)		7.08 (4.85)	10.25 (6.40)	<0.001	7.15 (4.74)	9.77 (5.46)	<0.001	NA
Intraoperative fentanyl consumption (mcg), mean (SD)		108.70 (38.59)	146.29 (59.97)	<0.001	108.66 (39.11)	130.45 (52.11)	<0.001	NA
Intraoperative epidural analgesia, n (%)		1 (0.3)	25 (1.6)	0.045	1 (0.3)	6 (1.8)	0.057	NA
Intraoperative NSAID administration, n (%)		86 (23.1)	309 (19.8)	0.155	73 (21.7)	66 (19.6)	0.505	NA
Intraoperative fluid intake (mL), mean (SD)		317.50 (192.21)	656.76 (617.77)	<0.001	319.52 (196.31)	539.02 (411.37)	<0.001	NA
Intraoperative blood loss (mL), mean (SD)		26.59 (72.34)	59.61 (203.22)	<0.001	27.29 (76.09)	40.39 (102.95)	0.061	NA
Intraoperative transfusion, n (%)		3 (0.8)	41 (2.6)	0.034	3 (0.9)	8 (2.4)	0.128	NA
Intraoperative adverse events, n (%)								
	Patient movement	61 (16.4)	0 (0.0)	<0.001	56 (16.7)	0 (0.0)	<0.001	NA
	Pulmonary episodes	17 (4.6)	42 (2.7)	0.059	16 (4.8)	10 (3.0)	0.230	NA
	Cardiovascular episodes	9 (2.4)	26 (1.7)	0.328	9 (2.7)	5 (1.5)	0.280	NA
**Postoperative variables**								
Grade of surgical complications, n (%)				0.472			0.060	NA
	0	121 (32.5)	459 (29.4)		110 (32.7)	108 (32.1)		
	I	247 (66.4)	1062 (68.1)		223 (66.4)	219 (65.2)		
	II	3 (0.8)	19 (1.2)		3 (0.9)	1 (0.3)		
	III	0 (0.0)	1 (0.1)		0 (0.0)	0 (0.0)		
	IV	1 (0.3)	12 (0.8)		0 (0.0)	4 (1.2)		
	V	0 (0.0)	7 (0.4)		0 (0.0)	4 (1.2)		
Postoperative fentanyl consumption (mcg), mean (SD)		337.79 (274.63)	425.31 (309.74)	<0.001	336.68 (275.55)	387.51 (276.08)	0.017	NA
Postoperative adverse events, n (%)								
	POST	12 (3.2)	66 (4.2)	0.376	11 (3.3)	18 (5.4)	0.184	NA
	PONV	4 (1.1)	30 (1.9)	0.264	3 (0.9)	7 (2.1)	0.203	NA
	Pulmonary episodes	6 (1.6)	81 (5.2)	0.003	5 (1.5)	16 (4.8)	0.015	NA
	Cardiovascular episodes	2 (0.5)	31 (2.0)	0.053	1 (0.3)	10 (3.0)	0.006	NA
Postoperative mechanical ventilation, n (%)		2 (0.5)	88 (5.6)	<0.001	1 (0.3)	13 (3.9)	0.001	NA
Postoperative ICU admission, n (%)		4 (1.1)	90 (5.8)	<0.001	3 (0.9)	13 (3.9)	0.011	NA
Length of postoperative hospital stay (days), mean (SD)		4.13 (2.61)	5.90 (8.77)	<0.001	4.10 (2.65)	5.68 (10.88)	0.010	NA
Length of total hospital stay (days), mean (SD)		7.06 (3.89)	10.04 (10.33)	<0.001	7.02 (3.82)	9.30 (11.48)	0.001	NA
Patient satisfaction, n (%)				0.002			0.005	NA
	Very satisfactory	256 (68.8)	946 (60.6)		230 (68.5)	209 (62.2)		
	Satisfactory	116 (31.2)	591 (37.9)		106 (31.5)	118 (35.1)		
	Neutral	0 (0.0)	23 (1.5)		0 (0.0)	9 (2.7)		
Surgeon satisfaction, n (%)				0.103			0.404	NA
	Very satisfactory	268 (72.0)	1072 (68.7)		243 (72.3)	241 (71.7)		
	Satisfactory	102 (27.4)	457 (29.3)		92 (27.4)	91 (27.1)		
	Neutral	2 (0.5)	31 (2.0)		1 (0.3)	4 (1.2)		
Surgery cost (USD), mean (SD)		1929.42 (579.95)	2383.70 (842.89)	<0.001	1938.56 (584.7)	2003.61 (715.51)	0.197	NA
Anesthesia cost (USD), mean (SD)		244.21 (95.51)	320.83 (167.60)	<0.001	245.98 (98.81)	283.21 (126.17)	<0.001	NA
Total cost (USD), mean (SD)		7107.48 (2386.64)	9929.54 (8212.35)	<0.001	7097.54 (2401.09)	8957.03 (9401.49)	<0.001	NA

Notes: Data shown as mean ± SD or n (%). Grade of surgical complications: Clavien–Dindo classification. IA, intubated anesthesia; ICU, intensive care unit; NA, not applicable; NIA, non-intubated anesthesia; NSAID, nonsteroid anti-inflammatory drug; PONV, postoperative nausea and vomiting; POST, postoperative sore throat; SD, standard deviation; SMD, standardized mean difference; USD, United States dollar.

**Table 3:** Subgroup analyses of perioperative complications by calendar period, surgeon experience, procedural type and anesthesia technique.

Stratified Variable	Ventilation Strategy	Crude OR (95% CI)	*p* Value	*p* Value (Interaction)	PS-Matched OR (95% CI)	*p* Value
*Calendar*				0.102		
*period*						
2015–2018	IA	1.00			1.00	
	NIA	1.17	0.637		1.21	0.627
		(0.61–2.24)			(0.57–2.57)	
2019–2022	IA	1.00			1.00	
	NIA	0.56	0.052		0.67	0.367
		(0.31–1.01)			(0.29–1.59)	
*Surgeon*				0.123		
*experience*						
<10 years	IA	cannot converge			cannot converge	
	NIA					
≥10 years	IA	1.00			1.00	
	NIA	0.80	0.328		0.79	0.408
		(0.52–1.25)			(0.45–1.38)	
*Procedural*				0.536		
*type*						
Wedge resection	IA	1.00			1.00	
	NIA	0.68	0.124		0.81	0.494
		(0.42–1.11)			(0.45–1.47)	
Other procedures	IA	1.00			1.00	
	NIA	1.03	0.960		0.83	0.832
		(0.31–3.47)			(0.15–4.50)	
*Anesthesia*				0.463		
*technique*						
TIVA	IA	1.00			1.00	
	NIA	0.37	0.175		0.35	0.214
		(0.09–1.56)			(0.07–1.83)	
INHA	IA	1.00			1.00	
	NIA	1.19	0.796		2.23	0.473
		(0.31–4.56)			(0.25–20.0)	
Mixed anesthesia	IA	1.00			1.00	
	NIA	0.72	0.218		0.80	0.513
		(0.42–1.22)			(0.42–1.55)	

CI, confidence interval; IA, intubated anesthesia; INHA, inhalational anesthesia; NIA, non-intubated anesthesia; OR, odds ratio; PS, propensity score; TIVA, total intravenous anesthesia.

**Table 4:** Logistic regression for unintended conversion to IA: univariable and multivariable models for VATS patients receiving NIA.

Variables		Univariable		Multivariable	
		OR (95% CI)	*p* Value	OR (95% CI)	*p* Value
Time since the earliest included patient (yrs)		0.85 (0.70–1.03)	0.091		
Sex, female (ref: male)		2.03 (0.82–5.03)	0.124		
Age (yrs old)		1.02 (0.98–1.07)	0.354		
BMI (kg/m^2^)		1.10 (0.96–1.26)	0.168		
Cigarette smoking (ref: no)		1.88 (0.66–5.39)	0.240		
Alcohol consumption (ref: no)		1.63 (0.20–13.3)	0.648		
Charlson comorbidity index		1.21 (0.87–1.67)	0.259		
FEV_1_ (% predicted)		1.00 (0.98–1.03)	0.755		
DLCO (% predicted)		1.02 (1.00–1.05)	0.061		
ASA class (ref: I)					
	II	0.55 (0.07–4.60)	0.584		
	III	1.87 (0.22–16.2)	0.571		
Mallampati score, III (ref: I + II)		3.80 (0.77–18.6)	0.100		
Histological subtype, SCC (ref: adenocarcinoma)		1.15 (0.43–3.09)	0.774		
Lesion site (ref: RUL)					
	RML	2.41 (0.73–7.95)	0.150		
	RLL	0.16 (0.02–1.34)	0.092		
	LUL	0.63 (0.18–2.18)	0.470		
	LLL	0.43 (0.09–2.11)	0.300		
TNM stage (ref: CIS)					
	I	0.68 (0.18–2.52)	0.560		
	II	1.02 (0.24–4.30)	0.974		
Differentiation grade (ref: I)					
	II	0.33 (0.11–0.98)	0.046	0.37 (0.09–1.45)	0.153
	III	0.91 (0.30–2.74)	0.865	1.05 (0.25–4.51)	0.945
Surgeon experience, ≥10 (ref: <10 yrs)		0.67 (0.08–5.43)	0.708		
Procedural type (ref: wedge resection)					
	Segmentectomy	0.95 (0.12–7.48)	0.959		
	Lobectomy	3.79 (0.77–18.7)	0.102		
Anesthesiologist experience, ≥10 (ref: <10 yrs)		1.29 (0.29–5.73)	0.742		
Anesthesia technique (ref: TIVA)					
	INHA	0.35 (0.04–3.52)	0.375		
	Mixed anesthesia	1.10 (0.31–3.93)	0.878		
Baseline MBP (mmHg)		1.01 (0.98–1.04)	0.498		
Baseline HR (beat/min)		1.00 (0.97–1.04)	0.817		
Baseline SpO_2_ (%)		0.87 (0.78–0.97)	0.011	0.89 (0.80–1.00)	0.042
Induction time (mins)		0.94 (0.88–1.01)	0.087		
Intraoperative NSAID administration (ref: no)		0.35 (0.08–1.56)	0.170		
Intraoperative patient movement (ref: no)		0.55 (0.12–2.44)	0.433		
Intraoperative pulmonary episode (ref: no)		35.2 (11.4–108)	<0.001	43.0 (11.6–160)	<0.001
Intraoperative cardiovascular episode (ref: no)		29.0 (7.06–119)	<0.001	50.0 (9.62–259)	<0.001

Notes: Odd ratios in the multivariable analyses were adjusted by those variables having significance in the univariable analyses. ASA, American Society of Anesthesiologists; BMI, body mass index; CI, confidence interval; CIS, carcinoma in situ; DLCO, diffusing capacity of the lung for carbon monoxide; FEV_1_, forced expiratory volume in 1 s; HR, heart rate; IA, intubated anesthesia; INHA, inhalational anesthesia; LLL, left lower lobe; LUL, left upper lobe; MBP, mean blood pressure; NIA, non-intubated anesthesia; NSAID, nonsteroid anti-inflammatory drug; OR, odds ratio; RLL, right lower lobe; RML, right middle lobe; RUL, right upper lobe; SCC, squamous cell carcinoma; SpO_2_, oxygen saturation; TIVA, total intravenous anesthesia; TNM, tumor–node–metastasis; VATS, video-assisted thoracoscopic surgery.

## Data Availability

The data analyzed in this study are available from the corresponding author on reasonable request.
